# Emotional well‐being, spiritual well‐being and resilience of advanced clinical practitioners in the United Kingdom during COVID‐19: an exploratory mixed method study

**DOI:** 10.1111/jonm.13577

**Published:** 2022-03-23

**Authors:** Melanie Rogers, Angela Windle, Lihua Wu, Vanessa Taylor, Chris Bale

**Affiliations:** ^1^ University of Huddersfield Huddersfield UK; ^2^ University of Central Lancashire Preston UK

**Keywords:** advanced roles, burnout, emotional reactivity, nurse practitioners, stress

## Abstract

**Aim:**

To investigate the emotional and spiritual well‐being and resilience of advanced clinical practitioners during COVID.

**Background:**

Resilience is a protective factor for emotional and spiritual well‐being. The pandemic has taken a toll on health professionals due to significant physical and psychological pressures. The impact of COVID‐19 on well‐being and resilience of advanced clinical practitioners is not known.

**Method:**

Three validated scales assessed resilience, emotional and spiritual well‐being. Seven hundred and thirty‐four responses were analysed.

**Results:**

Participants have low levels of emotional and spiritual well‐being. Participants with higher levels of spirituality reported greater resilience and those with higher levels of resilience reported greater well‐being.

**Conclusion:**

Advanced clinical practitioners' emotional and spiritual well‐being and resilience has been impacted significantly during the pandemic. Interventions are needed at team, service and systems levels to enhance well‐being and resilience.

**Implications for Nursing Management:**

Worryingly low levels of well‐being and resilience in advanced clinical practitioners have been found; support to increase well‐being and resilience is needed. Our findings can inform policies, resources and interventions aimed at enabling positive adaptation and enhanced resilience. Understanding and responding to the scale and impact of COVID‐19 on health care workers has become a key government recommendation following the pandemic.

## 1 BACKGROUND

The COVID‐19 pandemic has had a devastating impact globally including unprecedented deaths of health care workers globally (Jackson et al., 2020). The limited pandemic planning in the United Kingdom led to insufficient supplies of personal protective equipment (PPE), workforce and resource shortages, adding to the stress and pressure health care workers have faced (Jackson et al., 2020). It is, therefore, unsurprising that there has been significant toll on health care workers in terms of absenteeism, sickness and mental health problems (Wood et al., 2021). A systematic review focused on the mental health and well‐being of nurses and midwives in the United Kingdom published before the COVID‐19 pandemic identified these professionals were already at considerable risk of stress, burnout and mental health problems (Kinman et al., 2020). COVID‐19 has exacerbated these stressors with reports of the highest levels of sickness amongst health service staff since 2009 (Twinch, 2020). Health care workers have faced situations that have led to extreme stress, impossible decisions and working with scant resources. As part of the workforce, the coping strategies of advanced clinical practitioners (ACPs) have also been tested, and it is likely that their mental health has been adversely affected (Duncan et al., 2021).

Advanced practice developed through nursing in the United Kingdom. Historically advanced practice roles have been driven locally and have been in response to local health care and workforce needs. In 2017, health policy identified that advanced practice roles be standardized under the title “advanced clinical practitioner” (Health Education England [HEE], 2017). ACPs are multi‐professional health care practitioners educated to an advanced level of practice academically and clinically. The role incorporates four pillars of practice, namely, clinical, leadership and management, education and research (HEE, 2017).

In an online workshop exploring participant's (*n* = 1000) perceptions about the extent to which the full scope of ACP services had been used during the pandemic, 85% of participants reported that ACPs had made a useful contribution to the pandemic response, while 52% felt that these roles were not fully utilized (Health Education England, 2020). This under‐utilization may be because the ACP role is not fully understood by policy makers and employers (Health Education England, 2020).

The pandemic response required many ACPs to be redeployed into critical care areas to support direct patient care delivery. Others provided clinical assessment and management in community services for patients being cared for at home or in residential settings. Kang et al. (2020) identified significant stressors for health care workers during pandemics including redeployment, witnessing death and suffering on a large scale, risk of virus transmission at the workplace and at home to loved ones (Kang et al., 2020). Coping with the impact of these stressors during a pandemic requires resilience to protect the professional's mental well‐being (Pollock et al., 2020).

There is a close link between mental health and resilience (Hu et al., 2015). When exploring the well‐being of health staff during COVID‐19, Huffman et al. (2021) found resilience safeguarded against the negative psychological impact of stressors.

In a review of 56 papers focused on the mental health impact of health care workers during pandemics, Ricci Cabello et al. (2020) found that severe stress, burn‐out, reduced emotional well‐being and long‐term psychological damage are commonly reported. They also report that these factors led to an inability to meet the urgent needs posed by pandemics. Similarly, Rees et al. (2015) identified that reduced emotional well‐being of health care workers greatly increases absenteeism and can lead to burnout and compassion fatigue. This impacts on patient care and patient safety (Cheng et al., 2020).

Conceptualizations of resilience vary. Early definitions focus on the individual and view resilience as a trait people either have or do not have. These conceptualizations of resilience are reflected in various psychological measures including the Connor‐Davidson Resilience Scale (Connor & Davidson, 2003). Later theories emphasized that resilience can be learnt and is modifiable. Turner (2014) defined resilience as the capacity to recover from adverse circumstances and as a protective factor against stress. One of the characteristics of innate resilience identified is the individual's spirit—the inner being of the individual. Spirituality has been described as innately human and embraces issues of meaning, purpose and hope (Rogers et al., 2020; Wattis et al., 2017). Health care workers who are aware of their own spiritualty and have higher levels of spiritual well‐being are more likely to be more resilient (Meybodi & Mohammadi, 2020). Spirituality has been shown to have a protective impact on resilience (Sharma et al., 2017; Walsh, 2019).

The Cyclic Resilience Development Model (Grafton et al., 2010) identified resilience as a resource that individuals can draw to cope more effectively during stressful situations (positive adaptation) and to use the situation as a learning experience to restore and strengthen the biopsychosocial‐spiritual well‐being of the self and reduce vulnerability to future stress through greater resilience (cognitive transformation) (Jackson et al., 2007). In contrast, a reduction in resilience leads to burnout which in turn impacts workforce capacity and patient care (Eley et al., 2013).

These definitions and conceptualizations, which indicate the relationship which exists between resilience and biopsychosocial‐spiritual well‐being, informed this study focused on ACPs. While there has been rapid growth in research examining the impact of COVID‐19 on emotional well‐being and the psychological impact of COVID‐19 on health professionals (Mojtahedi et al., 2021), no studies have examined the emotional and spiritual well‐being and resilience of ACPs. Identifying these is critically important as the findings can inform policies, resources and interventions aimed at building greater ACP workforce resilience.

The aim of this study was to identify the impact of the COVID‐19 pandemic on the emotional and spiritual well‐being and resilience of ACPs across the United Kingdom.

## 2 METHODS

This exploratory mixed method web‐based study focused on a cross section of UK ACPs over a 2 month period in 2020. It was designed to determine participants' emotional and spiritual well‐being and resilience in relation to their experiences during the COVID‐19 pandemic. This paper reports the quantitative findings. Demographic data and exposure to COVID are also reported. The STROBE criteria were applied and followed for this study.

## 3 DESIGN AND DATA COLLECTION

Three validated scales were used to assess ACPs emotional and spiritual well‐being and resilience. These scales were the Warwick‐Edinburgh Mental Well‐being Scale, FACIT‐12 Spiritual Well‐Being Scale and Connor‐Davidson Resilience 10 Scale.

Emotional well‐being was measured using the Warwick‐Edinburgh Wellbeing Scale (WEMWBS) (NHS Scotland, 2006). The WEMWBS was developed to measure mental well‐being in the general population, as well as being used to demonstrate outcomes for research projects. The scale is validated for use with adults over 16 years and consists of 14 five‐point Likert‐scale questions related to participants' thoughts and feelings during the previous 2 weeks. The total scores range from 14 to 70, with higher scores indicating a more positive sense of emotional well‐being. Scores below 42 are considered to correspond to low well‐being, whereas scores above 60 correspond to high well‐being. The scale has been extensively used and validated across a range of populations (Stewart‐Brown et al., 2011), and demonstrates good reliability, with a high level of internal consistency in our sample (Cronbach's α = .91).

The Functional Assessment of Chronic Illness Therapy Spiritual Well‐Being (FACIT‐12‐ version 4) is from a collection of scales developed to measure quality of life domains such as social well‐being, physical well‐being and spiritual well‐being in people over 18 years old (Peterman et al., 2014). Version 4 was utilized as this is relevant for health care workers rather than patients. The FACIT‐12 was used for this study and scoring remained consistent with commonly observed scoring practices of this scale (Bredle et al., 2011). The FACIT‐12 survey consists of 12 questions scored on a Likert‐scale, eight questions relate to meaning and sense of peace, and four relate to faith. The FACIT 12 manual scoring guide was used with some items reverse scored. Higher scores indicate higher spiritual well‐being. Scoring for meaning, peace and faith may be reported separately acknowledging those who may not have a faith but describe themselves as ‘spiritual’. The FACIT‐12 has been validated and used in research across a range of populations and demonstrated an acceptable level of internal reliability in our sample (Cronbach's α = .69).

Resilience was measured using the Connor‐Davidson Resilience Scale. The original Connor‐Davidson Resilience (CD‐RISC) Scales were developed with 25 questions to measure resilience levels in patients with post‐traumatic stress disorder (Connor & Davidson, 2003). After factor analysis, the shorter 10 question scale (CD‐RISC10) was developed and has been used in multiple settings, including with health professionals (Connor & Davidson, 2003). The 10 questions on a 5‐point Likert‐scale relate to trait resilience and psychological resilience, and higher scores indicate higher levels of resilience. The CD‐RISC 10 has been extensively used and validated in a range of populations (Davidson, 2018), and it demonstrated a good level of internal consistency in our sample (Cronbach's α = .89).

Permission to use all three scales was given by the authors prior to the study.

An online Qualtrics survey incorporating these scales and demographic questions asked participants to indicate their gender, age, level of education and number of years of experience working as an ACP. Participants were also asked to indicate their professional title, work setting and working hours per week. Qualitative data were also collected via Qualtrics in the form of open responses and are reported separately. The survey was open from 1 July to 31 August 2020.

Snowball sampling was used drawing on the personal and professional advanced practice networks of the research team throughout the United Kingdom. Participants received an invitation and a survey link via email or social media. All responses were anonymous. Eligible participants:

- Were employed as an ACP and met the Health Education England (2017) definition of an advanced clinical practitioner: OR
- Were credentialed as an advanced practitioner by either the Royal College of Nursing or a national government body OR
- Were employed as a trainee ACP and were undertaking an advanced clinical practice master's degree.

Ethical approval was granted by the University of Huddersfield (SREIC 2021/043).

## 4 DATA ANALYSIS

Analysis was conducted following the instruction guidance developed by the scale authors. Quantitative analysis was completed by the statistician supported by the principal and co‐investigators. Six hundred and three surveys were completed in full. One hundred and thirty‐one participants (21.7%) had one or more items of missing data. However, the only variables where more than 5% of participants had missing data were those asking about experiences with COVID‐19 (Table 2). Missing values were not imputed, and participants with any missing data for a given variable were excluded from any analyses involving that variable.

Descriptive statistics were calculated for all study variables and presented in Tables 1 and 2. To assess the overall level of emotional well‐being, that is, the total WEMWBS scores in the current sample, a one‐sample *t* test comparing this with a previous general population sample taken from the Health Survey for England 2011) was conducted, and the size of this difference was assessed using Hedges' *g* (Hedges, 1981). Several one‐way ANOVAs were conducted to examine whether levels of emotional well‐being, resilience (CDRISC‐10), overall spirituality (FACIT‐SP‐12), faith and meaning differed between participants working in different clinical roles and settings, with different levels of highest qualification, and who worked different numbers of hours per week.

**TABLE 1:** Descriptive statistics for all study measures by demographic group

Variable		*N* (%)	WEMWBS		CDRISC		FACIT‐SP‐12 Total		FACIT‐SP‐12 faith		FACIT‐SP‐12 meaning	
			M	SD	M	SD	M	SD	M	SD	M	SD
Sex	Male	71 (12)	47.6	8.6	28.2	4.4	37.0	8.7	7.9	3.5	25.8	3.7
	Female	531 (88)	44.8	7.9	27.6	5.9	34.8	7.8	7.1	2.8	24.9	3.8
Title	Advanced clinical practitioner	344 (57)	45.4	8.1	28.4	5.7	35.0	7.8	7.1	2.8	25.0	3.6
	Nurse practitioner	95 (16)	43.9	8.3	25.7	6.5	33.9	8.4	7.0	2.9	24.6	4.2
	Clinical nurse specialist	26 (4)	46.4	7.5	27.1	4.3	37.2	8.7	8.8	3.7	24.9	4.5
	MSc ACP student	121 (20)	45.1	7.9	27.4	5.5	35.8	7.7	7.2	2.9	25.4	3.7
	Other	12 (2)	42.9	7.9	27.0	3.6	34.6	9.5	8.7	3.3	24.9	4.3
Education	Diploma	34 (6)	44.4	7.6	26.2	7.9	35.6	7.6	7.7	3.0	25.1	3.8
	Bachelor's degree	136 (23)	44.6	8.3	27.1	5.8	35.0	8.8	7.4	3.1	24.9	3.9
	Master's degree	402 (67)	45.2	8.1	28.0	5.6	35.1	7.8	7.1	2.9	25.0	3.8
	Doctoral degree	7 (1)	47.3	6.1	28.6	4.0	33.5	5.3	5.5	0.5	25.3	4.0
	Other	24 (4)	46.0	6.5	26.9	5.6	35.5	6.7	7.5	2.7	25.3	2.9
Clinical setting	Primary care	375 (62)	45.0	8.2	27.4	6.1	34.8	8.0	7.2	2.9	24.9	3.9
	Secondary care	136 (23)	45.6	7.9	28.3	5.6	36.1	8.2	7.6	3.1	25.4	3.6
	Long‐term care/nursing home	4 (1)	42.5	5.2	23.7	3.8	33.5	4.5	6.8	3.3	24.8	1.5
	Intensive care/emergency department/COVID ward	58 (10)	44.4	7.6	27.8	4.1	34.5	7.0	6.5	2.2	24.8	3.6
Work hours/week	None currently	2 (1)	53.0	8.5	32.0	7.1	42.0	14.1	10.0	7.1	29.0	5.7
	1–12	8 (1)	50.1	7.7	30.6	5.3	37.4	10.7	7.9	4.2	25.3	3.9
	13–24	52 (9)	45.0	8.1	26.6	6.2	35.9	7.6	7.8	3.0	25.0	4.2
	25–36	173 (29)	44.4	8.1	27.2	5.9	35.0	7.8	7.2	3.0	24.8	3.5
	37–48	356 (59)	45.2	8.0	27.9	6.0	35.0	8.0	7.1	2.8	25.1	3.8
	>48	12 (2)	45.0	8.5	27.5	4.5	33.4	8.2	6.3	3.0	24.2	5.1
Total		603	45.1	8.1	27.6	6.0	35.1	7.9	7.2	2.9	25.1	3.8

Abbreviations: CDRISC, Connor‐Davidson Resilience Scale; FACIT‐SP‐12, Functional Assessment of Chronic Illness Therapy – Spiritual Well‐Being 12 Item Scale; WEMWBS, Warwick‐Edinburgh Mental Wellbeing Scale.

**TABLE 2:** Experiences with COVID‐19, mental well‐being, resilience, and spirituality

Question		*N* (%)	WEMWBS		CDRISC		FACIT‐SP‐12 Total		FACIT‐SP‐12 faith		FACIT‐SP‐12 meaning	
			M	SD	M	SD	M	SD	M	SD	M	SD
Are you providing direct care or testing for patients with COVID‐19?	No	193 (41)	46.1	7.4	27.6	6.3	35.9	7.8	7.5	3.0	25.2	3.9
	Yes	279 (59)	44.7	8.1	27.7	5.4	34.6	7.7	7.0	2.7	25.0	3.6
Have you had experience providing care for patients during previous epidemics (for example, Ebola, SARS, MERS)?	No	317 (67)	45.5	7.6	27.5	5.9	35.3	7.7	7.3	2.8	25.2	3.5
	Yes	156 (33)	44.7	8.3	28.0	5.6	34.8	8.0	7.0	2.7	25.0	4.2
Have you been diagnosed with COVID‐19?	No	430 (91)	45.4	7.9	27.6	5.9	35.1	7.7	7.1	2.8	25.1	3.7
	Yes	43 (9)	44.3	7.2	28.5	5.3	35.1	8.9	7.7	3.2	24.9	4.0
Have any of your family members or your co‐workers been diagnosed with COVID‐19?	No	181 (38)	44.5	7.9	27.3	6.5	34.3	8.1	6.8	2.7	24.6	4.0
	Yes	292 (62)	45.7	7.8	27.9	5.3	35.7	7.5	7.4	2.9	25.4	3.5

Abbreviations: CDRISC, Connor‐Davidson Resilience Scale; FACIT‐SP‐12, Functional Assessment of Chronic Illness Therapy – Spiritual Well‐Being 12 Item Scale; WEMWBS, Warwick‐Edinburgh Mental Wellbeing Scale.

A stepwise linear multiple regression analysis was conducted to examine potential predictors of emotional well‐being in the current sample. Whether resilience and spirituality predicted emotional well‐being after controlling for other relevant variables. In the first step, the continuous variables age and the number of years participants had worked, and categorical variables were entered. The categorical variables used were as follows: (dummy coded) sex, whether participants were providing direct care or testing for COVID‐19, whether they had previous experience providing care in epidemics and whether they or any of their family or co‐workers had been diagnosed with COVID‐19. In the second step, resilience, faith and meaning were entered as additional predictors. A similar analysis was conducted to examine potential predictors of resilience, following the same procedure, with the exception that in the second step, faith and meaning were entered as predictors. In order to examine potential predictors of spirituality, the predictor variables described for the first step above were simultaneously entered into a single multiple regression model.

The alpha level for all analyses was set at .05, and any results with *p* values lower than this were considered to be statistically significant.

## 5 RESULTS

Table 1 shows the number of participants in different demographic and occupational groups. Our participants were aged between 27 and 67 (M = 45.8, SD = 8.7) and had worked as ACPs for between 1 and 24 years (M = 5.9, SD = 5.0). They were predominantly (88%) women, and most (62%) were employed in primary care, with the remainder working in secondary care (23%), intensive care, emergency departments and COVID wards (10%) and long‐term care and nursing homes (1%). The majority (57%) were advanced clinical practitioners (ACPs), though some were trainee ACPs studying for this role (20%) or identified themselves as nurse practitioners (16%), with relatively fewer clinical nurse specialists (4%). Most (67%) were educated to master's level, with the remainder holding bachelor's degrees (23%) or diplomas (6%). More than 60% report working more than 37 h per week.

Table 2 shows responses about their experiences during the pandemic. More than half (59%) were providing direct care or testing for patients with COVID‐19, although most (67%) had no experience of providing care in previous pandemics. Nine percent of participants had been diagnosed with COVID‐19, but the majority (62%) had family members or co‐workers who had been.

WEMWBS scores for our total sample were significantly lower than population norms taken from the Health Survey for England (2011), M = 45.1 versus 51.6, *t*(596) = −19.58, *p* < .001. This was a medium to large effect (Hedges' *g* = .75), which suggests that levels of emotional well‐being are significantly lower in the ACP sample than in the pre‐COVID‐19 general population. We also compared WEMWBS scores in our sample to an international general population sample surveyed in July 2020 (Foster et al., 2021) and found no significant difference between these samples, M = 45.1 versus 45.4, *t*(596) = −.83, *p* = .41, suggesting that overall levels of mental well‐being in our sample were the same as those of the wider population at this point in the pandemic.

Multiple regression analyses indicated that participant's age, sex and whether they were providing direct care or testing for COVID‐19 significantly predicted their levels of emotional well‐being, *F*(7, 440) = 3.83, *p* < .001, but accounted for only about 4% or the variance in this (adjusted *r*‐squared = .04). Adding spirituality and resilience to the model accounted for an additional 58% of the variance in this outcome variable. This indicates that, after controlling for demographic and occupational differences, and participants' experiences during the pandemic, spirituality and resilience strongly predicted emotional well‐being, *F*(3, 437) = 236.07, *p* < .001, *r*‐squared change = .58. However, while the meaning component of spirituality significantly predicted well‐being in this final model, the faith subscale did not.

These results suggest that male (β = .09, B = 2.31, 95% CI [.84, 3.8], *p* < .005) and older (β = .13, B = .12, 95% CI [.06, .18], *p* < .001) participants in our sample had significantly higher levels of emotional well‐being. Participants who were providing direct care or testing for COVID‐19 had significantly lower levels of emotional well‐being (β = −.07, B = −1.08, 95% CI [−2.01, −.16], *p* < .05). Participants who reported higher levels of resilience also reported greater emotional well‐being (β = .25, B = .34, 95% CI [.25, .43], *p* < .001). With respect to spirituality, although faith did not predict emotional well‐being in our sample, meaning was the strongest predictor of well‐being, with participants who reported higher levels of meaning also reporting significantly greater emotional well‐being (β = .58, B = 1.22, 95% CI [1.07, 1.37], *p* < .001).

In this study, there were no significant differences in emotional well‐being, resilience or spirituality between different demographic groups (Table 1), demonstrating similar levels across participants in different clinical roles and settings, with different qualifications, and with different work patterns. Multiple regression analyses indicated that the only significant predictor of resilience was spirituality, *F*(9, 442) = 23.47, *p* < .001, which accounted for 31% of the variance in resilience (adjusted *r*‐squared = .31). Participants with higher levels of faith (β = .10, B = .21, 95% CI [.03, .40], *p* < .05), and especially meaning (β = .51, B = .81, 95% CI [.67, .94], *p* < .001), also reported significantly greater levels of resilience.

The only significant individual predictors of spirituality were sex and having had a family member or co‐worker diagnosed with COVID‐19, but these accounted for less than 2% of the variance in spirituality (adjusted *r*‐squared = .015), and the overall regression model was only marginally significant, *F* (7, 446) = 1.96, *p* = .06. In the current sample, men reported higher levels of spirituality than women (β = .10, B = .72, 95% CI [−.14, .1.58], *p* < .05) and participants who had had a family member or co‐worker who had been diagnosed with COVID‐19 (β = .11, B = .68, 95% CI [.13, 1.22], *p* < .05), reported higher levels of spirituality than those who had not.

## 6 DISCUSSION

This paper reports the quantitative findings from a study of ACPs' emotional and spiritual well‐being and resilience 4 months after the first UK COVID‐19 outbreak. Regardless of their demographics (in the main), ACPs reported significantly lower levels of emotional and spiritual well‐being and resilience. Notably, the WEMWBS scores were significantly lower for these ACP participants than in the (pre‐COVID‐19) general population. Our findings support Kinman et al. (2020) who, in an evidence review on the mental well‐being of UK nurses and midwives, identified the high levels of work‐related stress, burnout and mental health problems which existed pre‐COVID. These authors also speculated that these are likely to have risen further during the pandemic, with staff at high risk of post‐traumatic stress symptoms and moral distress. The government have committed to develop a culture where staff health and well‐being is a focus embedded across all organisations, this commitment includes working with systems and managers to improve the day to day experience and well‐being of the workforce as the post COVID‐19 recovery phase begins (HM Government, 2022).

Our findings identify the low well‐being scores of ACPs and provide a baseline to repeat this survey to understand the on‐going impact of the COVID‐19 pandemic on ACPs well‐being. Kinman et al. (2020) also suggest that the prevalence of presenteeism is likely to have risen in the sector during the pandemic. More than 60% of our participants report working in excess of 37 h per week. This may be in response to staffing shortages and diminishing resources as practitioners strive hard to ensure that their working conditions and any stress they may be experiencing does not adversely affect their patients. It may be reasonable to assume that limited time away from work, alongside the impact of lockdown, has led to physical and emotional fatigue and exacerbating symptoms in those ACPs constantly exposed to stressors when providing care for people affected by COVID‐19, including the impact of redeployment or new ways of working, the witnessing of death and suffering on a large scale, the risk of virus transmission at the workplace and at home to their loved ones (Kang, 2020).

Our findings suggest that caring for people with COVID‐19, or having a close family or co‐worker diagnosed with COVID‐19 (62%), influenced the spiritual well‐being of ACPs, particularly the meaning component. Meaning was the strongest predictor of emotional well‐being. Finding meaning in their work is considered essential to give nurses the strength to carry on amid very demanding environments (Malloy et al., 2015). For some, redeployment required the forming of relationships with new clinical teams. New ways of working, including the need for PPE, social distancing and remote consultations may have also affected ACPs abilities to make connections and develop relationships with patients and families. Caring for people with COVID‐19 also led to challenging end of life decision‐making that demands a deep consideration and respect for patient and family. Kinman et al. (2020) identified particularly high risk of moral distress if institutional pressures and constraints stop professionals from pursuing what they believe to be the most appropriate care or course of action for their patients. Moral injury can occur when individuals act against their moral conscience and values, such as imposed visiting restrictions. Undertaking roles outside of their usual service may also impact on emotional well‐being and meaning as ACPs are required to practice outside their zone of confidence or expertise (Twinch, 2020). Sixty seven percent (67%) of our participants report not having had experience of providing care during previous pandemics.

The importance of a ‘mentoring culture’ and organisational support has been identified as contributing to ‘meaning making’ and facilitating a supportive work environment (Malloy et al., 2015). Our findings suggest that meaning making and a sense of purpose as care providers may have facilitated positive adaptation and cognitive transformation to cope effectively during these stressful situations (Grafton et al., 2010). Indeed, HEE (2020) reported that the impact of COVID‐19 on the working lives of ACPs is stark with over 90% stating that the COVID‐19 response has changed the way they work to some extent. Although the impact on their working lives has been significant, over 70% felt that the response had created opportunities for them to develop their skills and knowledge.

Modelling of our participants' responses revealed a gender difference in resilience and well‐being with male ACPs reporting higher levels of meaning and emotional well‐being. With only 12% (*n* = 71) male respondents, these results should be viewed with caution and should be investigated further. The other significant factor was age. Older ACPs reported higher levels of emotional well‐being than younger ACPs. This finding may be a consequence of the mandated limited social contact experienced by younger people during COVID‐19 (Bu et al., 2020). Our findings may also be informed by Cai et al. (2020) who compared experienced with inexperienced frontline workers and found that inexperienced workers scored lower on total resilience on the Connor‐David resilience scale and had more mental health symptoms. While our participants in ACP roles are experienced health care professionals, in this context, the impact of being redeployed to other clinical areas and providing care during a pandemic may have contributed to these participants viewing themselves as ‘inexperienced’.

Focusing solely on the resilience of individuals is reported to divert attention from the collective responsibility of society to protect individuals. This means that failure to cope with challenges is considered a failure of the individual, who is viewed as not having developed sufficient resilience, rather than considering contextual factors. Masten (2015) defined resilience as the capacity to adapt positively and successfully to challenging circumstances or adversity, describing how this capacity manifests at various levels including individuals, families, and communities. Southwick et al. (2014) described how determinants of resilience include a host of biological, psychological, social, and cultural factors that interact with one another to determine how one responds to stressful experiences. As highlighted by Malloy et al. (2015), a ‘mentoring culture’ is an identified theme which contributes meaning to nurses work alongside relationships, compassionate caring an identity. While our focus here has been reporting the data from these well‐being scales, we recognize resilience is also influenced by various external and environmental factors which are captured in the qualitative data we gathered. Southwick et al. (2014) stated ‘it is critical to understand that humans are embedded in families, families in organisations and communities, and communities in societies and cultures’ and that their resilience will be affected by factors at each of these levels. Therefore, the link between resilience and workplace culture should also be considered when identifying what can be done individually and collectively to contribute to resilient health care teams and organisations.

In the United Kingdom, initiatives have been implemented which attempt to mitigate the effects of the pandemic on health care practitioner's well‐being (NHS, 2021) yet the long‐term impact on well‐being and resilience is currently unknown. Greenberg et al. (2020) recommend that to prevent long term mental health problems and moral injury all health care workers should be prepared for the moral dilemmas they will face during a pandemic with ongoing support and meaningful narratives which may help individuals to process and reflect on the trauma experienced. The government has highlighted that the potential longer‐term effects of COVID‐19 on the workforce now requires an operational approach to support staff recovery, as their health and well‐being is a major factor in retention of an experienced workforce to deliver a sustainable service (HM Government, 2022).

Although our study design has enabled us to understand some of the factors that have impacted ACPs emotional and spiritual well‐being and resilience, there are limitations to this study. This study was conducted during the first national lockdown and, as such, is a snapshot in time. The respondents self‐selected and self‐reported. This means they may have only shared what they felt comfortable reporting and sharing. In addition, due to the study design it is a challenge to establish any causal relationships from the findings. Finally, as the COVID‐19 crisis has had such a significant impact on practitioners, and we asked for their response's mid pandemic, recall bias may be present. A repeat of this study was conducted in late 2021 to collect longitudinal data which will help to better understand the true extent of how COVID‐19 has impacted ACPs in the United Kingdom. Heterogeneity of the sample could also be improved as most of the participants were female and from a nursing background.

## 7 CONCLUSION

The challenges facing health care workers globally has been unprecedented. Research highlighting the frontline experiences of health care staff during COVID‐19 reports the anxiety and stress they experience. ACPs in the United Kingdom have played an integral role in providing care to patients in challenging situations. These challenges have been complex and variable impacting their emotional and spiritual well‐being and resilience. Our study builds on the evidence to establish a baseline of the initial impact on well‐being, spirituality and resilience on ACPs.

It is concerning to see levels of resilience and well‐being are lower than pre‐COVID‐19 results. The impact of these challenges on well‐being and resilience has significant implications for staff retention. There is now a need to consider how to improve ACPs emotional and spiritual well‐being and resilience to prevent long‐term implications and an adverse impact on patient care.

## 8 IMPLICATIONS FOR NURSING MANAGEMENT

Nurse managers need to monitor the wellbeing of their employees to prevent a further crisis in the healthcare system. This study has significant implications for nursing management who can respond with changes in policy, practice, research and education to support their staff. Recovery of the workforce will need embedded change and a preventative approach for staff well‐being and health, to attract and retain those members of the workforce that are highly skilled and experienced such as ACPs.

## CONFLICT OF INTEREST

The authors declare that they are no conflicts of interests that have influenced the work reported in this paper.

## ETHICS STATEMENT

Ethic approval was given by the University of Huddersfield. Reference number: SREIC/2020/039 on 14 May 2020.

## Data Availability

The data that support the findings of this study are available on request from the corresponding author. The data are not publicly available due to privacy or ethical restrictions.
